# Plasma Levels of Decorin Increased in Patients during the Progression of Breast Cancer

**DOI:** 10.3390/jcm10235530

**Published:** 2021-11-26

**Authors:** Tokuko Hosoya, Goshi Oda, Tsuyoshi Nakagawa, Iichiroh Onishi, Tadashi Hosoya, Megumi Ishiguro, Toshiaki Ishikawa, Hiroyuki Uetake

**Affiliations:** 1 Department of Breast Surgery, Tokyo Medical and Dental University, 1-5-45 Yushima, Bunkyo-ku, Tokyo 113-8519, Japan; oda.srg2@tmd.ac.jp (G.O.); nakagawa.srg2@tmd.ac.jp (T.N.); 2 Department of Pathology, Graduate School of Medicine and Dentistry, Tokyo Medical and Dental University, 1-5-45 Yushima, Bunkyo-ku, Tokyo 113-8519, Japan; iichpth2@tmd.ac.jp; 3 Moores Cancer Center, University of California San Diego, 3855 Health Sciences Drive, La Jolla, CA 92037, USA; hosorheu@tmd.ac.jp; 4 Department of Specialized Surgeries, Graduate School of Medical and Dental Sciences, Tokyo Medical and Dental University, 1-5-45 Yushima, Bunkyo-ku, Tokyo 113-8519, Japan; ishiguro.srg2@tmd.ac.jp (M.I.); ishi.srg2@tmd.ac.jp (T.I.); h-uetake.srg2@tmd.ac.jp (H.U.)

**Keywords:** decorin, breast cancer, proteoglycan, extracellular matrix, cancer stroma

## Abstract

Decorin (DCN), an extracellular matrix proteoglycan found in tumor surrounding tissues, is a natural inhibitor of tumor cell proliferation and invasion. We conducted a cross-sectional observation study to evaluate the association of the pathological stage with the levels of DCN in plasma or tumor surrounding tissue. Among 118 patients who underwent breast surgery, 35 were designated as carcinoma in situ (Stage 0), 39 were Stage I, and 44 were Stage II or III. The stromal expression of DCN was quantified using a semiquantitative digital image analysis after immunohistochemical staining. The concentration of DCN was evaluated with a specific ELISA. As we have previously shown, stromal DCN expression was attenuated in the patients with Stage I, whereas stromal and plasma DCN was elevated paradoxically in those with Stage II/III. The elevated plasma DCN is an independent predictive factor of Stage II/III by the multivariate logistic regression analysis. The plasma level of DCN was negatively correlated with stromal DCN expression only in patients with advanced disease (Stage II/III). The plasma level of DCN could become a useful biomarker for patients in the advanced stages. Extensive studies and further assessments are warranted for evaluating the prognostic significance and tumor characteristics to understand the clinical significances of stromal and systemic DCN.

## 1. Introduction

Decorin (DCN) is a small leucine-rich proteoglycan (SLRP) that is synthesized primarily by fibroblasts and myofibroblasts [1]. It is a component of the extracellular matrix (ECM) that provides structural support to cells and has a role in regulating cell proliferation, differentiation, and wound healing. DCN interacts with transforming growth factor-β (TGF-β) [2] and affects several pathways, including the epidermal growth factor receptor (EGFR), insulin-like growth factor receptor 1 (IGF-1R), hepatocyte growth factor receptor (Met), and Toll-like receptors (TLRs) [1,3].

We previously discovered that stromal DCN expression was significantly lower in the tumor-surrounding tissues of patients with invasive breast cancer (IBC) than in those with benign tumors or ductal carcinoma in situ (DCIS) [4]. Furthermore, its expression was attenuated in the invasive components rather than the DCIS components, even in the same subject. These findings suggested the possibility that the downregulation of stromal DCN expression may be involved in breast cancer progression. 

Several studies have shown that stromal DCN expression is primarily reduced or even suppressed by cancer cells in various epithelial cancers such as lung, colon, esophageal and oral cancer, and myeloma [5,6,7,8,9]. Therefore, the expression levels of DCN in cancer tissue have been proposed as a potential diagnostic or prognostic biomarker [9,10,11,12]. In this regard, DCN is considered as a potent natural inhibitor of tumor cell proliferation and invasion, whereas the other proteoglycans, namely, versican and biglycan, are not [3,13].

Soluble DCN is released from its binding partners in the ECM, including collagen type-I [14], and is measurable in the plasma while remaining active as a ligand. A few reports describe the association between the blood levels of DCN and specific cancer types or clinical characteristics [15,16,17,18]. However, the clinical significance was still unresolved in various cancers, including breast cancer. 

This study is designed to investigate the diagnostic role of the plasma DCN levels and evaluate any association with expression levels in cancer tissue, the progression of the disease, and the characteristics of the patients.

## 2. Materials and Methods

### 2.1. Study Subjects

This single-center cross-sectional observation study included 118 patients who underwent breast surgery between June 2012 and December 2014 in the Department of Breast Surgery at Tokyo Medical and Dental University (Tokyo, Japan). Among the patients, 83 were diagnosed with invasive breast cancer (IBC) and 35 with carcinoma in situ only. Mean age was 57.7 years (range, 26–89 years). None of the patients had distant metastasis. All specimens were formalin-fixed and paraffin-embedded. Additionally, plasma was corrected from 12 healthy volunteers and 14 patients with benign breast tumor as a reference. The characteristics of the patients were collected from electronic medical records. The Institutional Review Board approved the study (M2000-831 and M2000-964) and written informed consent was obtained from each patient before surgery.

### 2.2. Examination of Clinicopathological and Biological Features

After tissue samples were stained with hematoxylin-eosin (H&E), histopathological staging was performed using the International Union Against Cancer (UICC) Tumor-Node-Metastasis classification criteria, 8th edition. The clinical stages were identified following with the pathological staging. Namely, Stage 0 meant noninvasive breast cancer, called carcinoma in situ: the cancer cells were confined to the ducts and had not spread to the surrounding tissue of the breast. In Stage I, the tumor size of the largest invasive component was 2 cm or less and no regional lymph node macrometastasis (larger than 2 mm) nor distant metastasis was identified. In Stage II/III, the tumor size was more than 2 cm or the cancer had spread to regional lymph node(s) but not to distant organs. In Stage IV, the cancer had spread to distant organs, indicating that patients could not undergo curative surgery and were not included in our study.

Expression of estrogen receptor (ER) and Human Epidermal Growth Factor Receptor 2 (HER2) was evaluated using immunohistochemistry. The status of each tumor with regard to ER expression was determined from the percentage of all cancer cells within a given tumor with positive nuclear staining; the cut off value was set at 10%. The HER2 status of each tumor was determined according to the ASCO guideline [19]. Nuclear atypia, mitotic count, and nuclear grade were determined according to the General Rules for Clinical and Pathological Recording of Breast Cancer, 17th edition, by the Japanese Breast Cancer Society.

### 2.3. DCN Immunohistochemical Staining

For immunohistochemical analyses, tissue sections (4 µm thick) were deparaffinized over the course of 20 min and four 10 min incubations in xylene. Tissue sections were rehydrated, and antigen retrieval was then performed by incubating the sections in 10 mmol/l sodium citrate buffer (pH 6.0) in a temperature-controllable microwave (MW) processor (MI-77; Azumaya Co., Tokyo, Japan) at 98 °C for 20 min. Endogenous peroxidase activity was blocked using a solution of 3% hydrogen peroxide in absolute methanol for 15 min. Sections were incubated with anti-DCN mouse monoclonal antibodies (ab54728, Abcam, Cambridge, UK) (1:500 dilution) and the sections were then beam irradiated with MW processor at 27 °C for 15 min. The Histofine Simple Stain MAX-PO (MULTI) (Nichirei, Tokyo, Japan) was used according to the manufacturer’s instructions to detect bound primary antibodies. Color development was carried out with DAB (3,3′-diaminobenzidine tetrahydrochloride; Nichirei) for 6 min at room temperature. The sections were then counterstained with Mayer’s hematoxylin.

### 2.4. Immunohistochemical Evaluation

**Figure 1:**
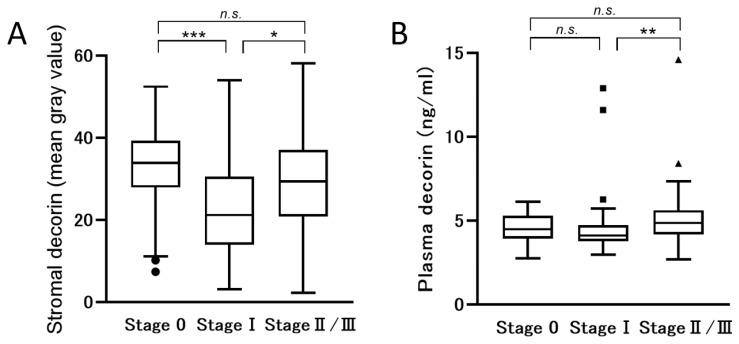
The plasma levels of DCN were elevated in the patients with advanced-stage breast cancer. (**A**): The quantified stromal expression of decorin was compared among the three groups, including clinical stage of carcinoma in situ (Stage 0), Stage I, and Stages II and III. (**B**): The concentration of decorin in plasma was similarly compared. Data were analyzed using one-way ANOVA and post hoc Holm–Bonferroni method in Figure A and using Kruskal–Wallis test and post hoc Steel–Dwass test in Figure B. Data showed box-and-whisker plot and *, **, *** indicated statistically significant *p* < 0.05, <0.01, and <0.001, respectively.

The immunostaining of DCN was analyzed under a light microscope (DP73, Olympus Corporation, Tokyo, Japan) using cellSens software (version 1.6, Olympus corporation, Tokyo, Japan). The evaluation of stromal DCN expression was performed around malignant cells using semiquantitative digital image analysis. The intensity of DCN signal was evaluated using the ImageJ software (Version 1.52o, U.S. National Institutes of Health, Bethesda, MD, USA), according to the method described as previously [4]. Briefly, stained specimens were viewed using a light microscope, and areas at the periphery lesions of tumor were captured as digital images (1600 × 1200 pixels) with a digital camera. For each digital image, the signal was digitized into a grayscale ranging from 0 (white) to 255 (black) (Figure 1A). The intensities of stromal DCN signal were standardized as mean gray value by subtracting the those of internal control (samples without primary antibody).

### 2.5. Enzyme-Linked Immunosorbent Assay

DCN levels in plasma of all the patients were measured using DuoSet enzyme-linked immunosorbent assay (ELISA) kit (R&D Systems, Minneapolis, MN, USA) according to the manufacturer’s instructions.

### 2.6. Statistical Analysis

Data were statistically analyzed using EZR (Division of Hematology, Saitama Medical Center, Jichi Medical University, Saitama, Japan), a free software for using R on graphical user interface [20]. Clinical findings among patients with different pathological stages were compared using one-way analysis of variance following post hoc Holm-Bonferroni or Kruskal-Wallis test following post hoc Steel-Dwass test depending on the data distribution. Spearman’s rank correlation was examined to identify the association of stromal expression and plasma concentration of DCN. Univariate and multivariate logistic regression analyses examined the effects of independent variables on the advanced stage to age, stromal expression of DCN, and plasma concentration of DCN. Cutoff points of each variable were chosen as median value in the patients with advanced stage. The odds ratio (OR), including 95% confidence interval (CI), were estimated.

## 3. Results

This section may be divided by subheadings. It should provide a concise and precise description of the experimental results, their interpretation, as well as the experimental conclusions that can be drawn.

### 3.1. The Clinical Significance of DCN in Patients with Breast Cancer

We analyzed the stromal and systemic expressions of DCN and compared them to the pathological stages of the patients with breast cancer. Of 118 patients, 35 were determined to have noninvasive cancer (Stage 0), 39 were Stage I, and 44 were at an advanced stage (Stage II and III). The data distribution was significantly different in age, stromal DCN, and plasma DCN (Table 1). The mean and ± standard deviation of plasma DCN in reference was 4.74 ± 1.63.

### 3.2. The Paradoxical Expression of DCN in Patients with Advanced Stage Disease

Since we identified that stromal DCN was attenuated in the surrounding tissue of tumors with invasive components, we analyzed the correlation between the stromal expression and plasma concentration of DCN to the different stages of breast cancer. Consistent with our previous report, the expression of stromal DCN was attenuated in breast cancer with invasive components even when the disease was still early (Figure 1A). Interestingly, stromal DCN was paradoxically elevated in the patients with an advanced stage (Stage II and Stage III) compared to those with Stage I (Figure 1A).

The concentration of circulating DCN in the plasma was higher in the patients with advanced-stage cancer (Mean ± SD = 5.18 ± 1.90 ng/mL, *p* = 0.007) than those with Stage I or Stage 0 (Mean ± SD = 4.58 ± 1.96 ng/mL and 4.56 ± 0.83 ng/mL, respectively) (Figure 1B).

These findings suggested that the production of DCN was upregulated as the cancer progressed.

### 3.3. A Negative Correlation between Stromal DCN Expression and Plasma DCN Concentration in the Patients with Advanced-Stage Breast Cancer

**Figure 2:**
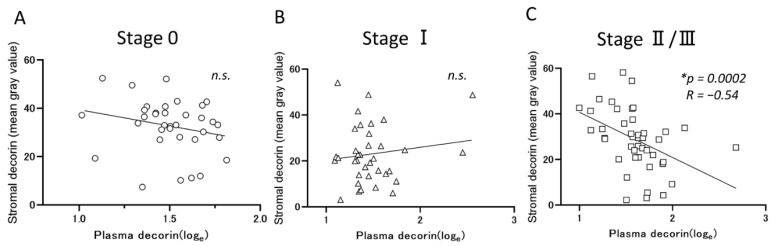
The plasma levels of decorin were negatively correlated with stromal decorin expression in the patients with advanced-stage cancer. (**A**–**C**): The correlation between stromal and plasma decorin was analyzed in patients with Stage 0 (**A**), Stage I (**B**), and Stage II and III (**C**) breast cancer. Data were analyzed using Spearman’s rank correlation and * indicated statistically significance.

Comparing the local expression and systemic levels of DCN, a negative correlation was observed only in the patients with advanced disease, and not in those with Stage 0 or Stage I (Figure 2A–C). These findings suggested the hypothesis that the elevation of plasma DCN was caused by a transfer of stromal DCN to the circulation, by the tumor-derived factors. Since the tumors in advanced stages obtained malignant characteristics, the inverse relationship of plasma and stromal DCN would be obvious in the patients with the advanced stage rather than the others.

### 3.4. The Elevation of Plasma Level of DCN Would Be an Independent Predictive Factor of the Advanced Stage

To identify predictive factors of the patients with the advanced stage, we analyzed clinical findings with univariate and multivariate logistic regression models. Multivariate logistic regression models revealed that the elevated plasma level of DCN (adjusted OR 2.50, 95% CI 1.09–5.69) and age (Adjusted OR 2.36, 95% CI 1.02–5.48) were identified as independent predictive factors of the advanced stage (Stage II/III) (Table 2).

### 3.5. No Histological Characteristics Were Associated with the Levels of Stromal rr Plasma DCN in Invasive Breast Cancer

To identify the factors associated with the expression or secretion of DCN, we compared the levels of stromal and plasma DCN to the histological characteristics in invasive breast cancer, including nuclear atypia, the mitotic count, ER status, and HER2 status. However, no association was identified among these factors and the levels of stromal DCN or plasma DCN (Table A1).

## 4. Discussion

This was the first study to show the association between the pathological stage and the levels of DCN in the circulatory or cancer-surrounding tissue in patients with breast cancer. As we have shown previously, stromal DCN expression was attenuated in the patients with Stage I, whereas stromal and plasma DCN was elevated paradoxically in those with Stage II/III. The elevated plasma DCN would be one of the independent predictive factors of Stage II/III. The plasma level of DCN was negatively correlated with stromal DCN expression only in patients with advanced disease, indicating that the plasma level of DCN increased as a consequence of the cancer progression via unknown tumor-derived factors.

The plasma level of DCN was increased and negatively correlated with the stromal DCN expression only in the patients with advanced-stage cancer, indicating that an increasing level of circulating DCN might suggest tumor progression. Since the sources of DCN in the tumor tissue are the tumor-surrounding fibroblasts and tumor-infiltrated inflammatory cells, changes in stromal and circulating DCN reflect the alteration of the tumor microenvironment. In cancer-associated fibroblasts (CAF), proteoglycan synthesis shifted from DCN to versican, biglycan, and type I collagen, resulting in tumor-supportive ECM formation, which allowed the spreading of the tumor [13]. In addition, CAF secreted several matrix metalloproteinases (MMPs), such as MMP-2 and MMP-8, which degraded DCN [21,22]. The altered function of CAF induced the transcription of DCN and the leakage into the circulation after degradation, resulting in the attenuation of local DCN and elevation of plasma DCN simultaneously, at least in some patients. Indeed, the expression of stromal DCN was attenuated in the myxoid periductal stromal architecture [23], produced by CAF [24,25]. Since the stromal expression of DCN exerted protective functions in tumor growth and tumor spreading [26,27], the local delivery of DCN could be a potent novel therapy for inhibiting tumor progression and spreading. In the preclinical models, the intra-tumor delivery of DCN using an adenoviral vector prevented tumor metastasis to the lung by reducing the ECM content [28] and the intravenous delivery of a DCN plasmid DNA prevented hepatocarcinogenesis [29]. Furthermore, it also overcame TGF-β-mediated immunosuppression, thereby inhibiting the accumulation of Treg cells and recruiting CD8+ T cells to the tumor. These findings suggested that the overexpression of DCN caused the tumor to become immunogenic [30].

Consistent with our previous report, the association of tumor invasiveness and the attenuated expression of DCN in the tumor-surrounding tissue were common findings in various cancers, including colon, lung, bladder, and esophageal cancers [5,6,7,8,9,16]. However, this study also demonstrated that circulating and locally expressed DCN was upregulated paradoxically as the stage progressed. In addition to the increased amount of transferring from stromal tissue, the transcription of DCN might be enhanced. Since cancer-associated soluble factors such as inflammatory cytokines and IGF-1, or nutrition deprivation increased the plasma levels of DCN [14,31,32], the upregulation of DCN could reflect the chronic inflammation or metabolic dysfunction observed with cancer progression at least partially. We must admit several limitations. Due to the cross-sectional observational nature of the study, the issue of residual confounding was unavoidable. The limited number of patients from a single center could cause a beta error in the analysis. Since we could obtain clinical samples from the patients who underwent curative surgery, our data missed the patients with further advanced disease. Although we did not evaluate the other proteoglycans, their concentrations in plasma and tissue should be analyzed since the dysregulation of DCN resulted in the formation of a tumor-supportive microenvironment. Additionally, enzymes such as MMP-2 and MMP-8 are worthy of analysis in order to understand their contribution to local and systemic DCN levels.

## 5. Conclusions

This study showed that plasma DCN levels were paradoxically elevated in patients with advanced-stage breast cancer despite the downregulation of local DCN in those with an early stage of invasive breast cancer. Further studies are required to identify the mechanism of plasma DCN elevation in the advanced-stage breast cancer. Although the diagnostic value of plasma DCN would be compared to conventional biomarkers in further study, plasma DCN levels could become a predictive factor of the advanced disease. Larger studies and further assessments of the prognostic significance of stromal and systemic DCN are warranted.

## Figures and Tables

**Table 1:** Comparison of stromal and plasma DCN, age, and biological features in different pathological stages.

Pathological Stage	Stage 0	Stage Ⅰ	Stage Ⅱ/Ⅲ	*p*-Value *
Number (*n*)	35	39	44	
Age (years)	55.2 ± 12.8	55.3 ± 12.7	61.5 ± 13.5	**0.048**
Stromal DCN (mean gray value)	32.7 ± 11.1	22.3 ± 12.2	28.9 ± 13.8	**0.003**
Plasma DCN (ng/mL)	4.56 ± 0.83	4.58 ± 1.96	5.18 ± 1.90	**0.008**

*: Data were analyzed using one-way ANOVA (age, plasma DCN) or Kruskal–Wallis test (stromal DCN) depending on the data distribution. Statistically significant *p* < 0.05 values are in bold. Data showed mean and ± standard deviation. DCN: decorin.

**Table 2:** Univariate analysis and multivariate logistic regression analysis associated with the advanced stage.

Variables	Univariate Analysis			Multivariate Analysis		
	OR	95% CI	*p*-Value	OR	95% CI	*p*-Value
Age	2.53	1.05–6.16	**0.025**	2.36	1.02–5.48	**0.045**
Stromal DCN	1.33	0.57–3.17	*0.555*	1.62	0.701–3.76	*0.259*
Plasma DCN	2.68	1.15–6.35	**0.017**	2.50	1.09–5.69	**0.030**

Statistically significant *p* < 0.05 values are in bold. OR: odds ratio; CI: confidence interval.

**Figure A1:**
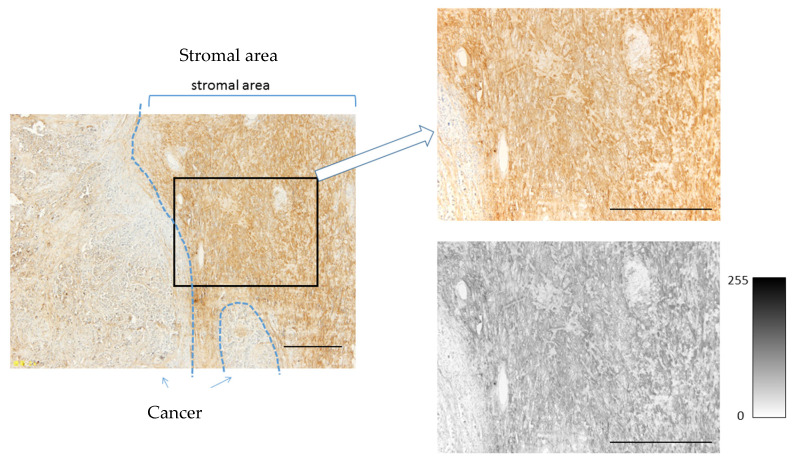
Digital image analysis of stromal DCN expression. The immunostained specimens were viewed using a light microscope, and stromal areas at the periphery of tumors were captured as digital images with a digital camera. For each digital image, the signal was digitized into a grayscale ranging from 0 (white) to 255 (black). Scale bars indicate 500 µm.

## Data Availability

The data that support the findings of this study are available from the corresponding author upon request.

## Appendix A

**Table A1:** The association of plasma decorin or stromal decorin and tumor characteristics in IBC.

	Status	Number	Plasma DCN Mean ± SD	*p*-Value ***	Stromal DCN Mean ± SD	*p*-Value
Nuclear atypia	1	5	5.2 ± 2.1		32.2 ± 13.7	
	2	56	5.0 ± 2.2		26.7 ± 14.2	
	3	21	4.7 ± 1.1	0.80	24 ± 11.3	0.44
Mitotic count	1	46	4.9 ± 2.1		27.2 ± 12.4	
	2	13	5.3 ± 2.5		29.1 ± 18.4	
	3	23	4.8 ± 1.1	0.67	23.1 ± 12	0.43
Nuclear grade	1	42	4.8 ± 2.2		27.2 ± 12.4	
	2	13	5.6 ± 2.5		30.6 ± 16.3	
	3	27	4.7 ± 1.1	0.39	23.1 ± 12.4	0.31
ER	Negative	11	4.7 ± 1.3		28.4 ± 16	
	Positive	72	4.9 ± 2	0.87	25.9 ± 13	0.63
HER2 score	0	43	4.8 ± 1.5		26.4 ± 11	
	1	17	5.3 ± 2.7		24.9 ± 11.6	
	2	12	5.3 ± 2.6		28.3 ± 20.1	
	3	11	4.3 ± 0.9	0.52	25.8 ± 17.3	0.87

*: Data were analyzed using one-way ANOVA (plasma DCN) or Kruskal–Wallis test (stromal DCN) depending on the data distribution. Data showed mean and ± standard deviation. DCN: decorin. ER: estrogen receptor. HER2: human Epidermal Growth Factor Receptor 2.
